# The Amino Terminus of LeuT Changes Conformation in an Environment Sensitive Manner

**DOI:** 10.1007/s11064-019-02928-9

**Published:** 2019-12-19

**Authors:** Jawad A. Khan, Azmat Sohail, Kumaresan Jayaraman, Dániel Szöllősi, Walter Sandtner, Harald H. Sitte, Thomas Stockner

**Affiliations:** 1 Institute of Pharmacology, Center for Physiology and Pharmacology, Medical University of Vienna, Waehringerstr. 13a, 1090 Vienna, Austria; 2 Institute for Pharmaceutical and Medicinal Chemistry, Heinrich Heine University Düsseldorf, Düsseldorf, Germany

**Keywords:** LeuT, Lanthanide resonance energy transfer, Simulations, Iodide quenching

## Abstract

Neurotransmitter:sodium symporters are highly expressed in the human brain and catalyze the uptake of substrate through the plasma membrane by using the electrochemical gradient of sodium as the energy source. The bacterial homolog LeuT, a small amino acid transporter isolated from the bacteria *Aquifex aeolicus*, is the founding member of the family and has been crystallized in three conformations. The N-terminus is structurally well defined and strongly interacts with the transporter core in the outward-facing conformations. However, it could not be resolved in the inward-facing conformation, which indicates enhanced mobility. Here we investigate conformations and dynamics of the N-terminus, by combining molecular dynamics simulations with experimental verification using distance measurements and accessibility studies. We found strongly increased dynamics of the N-terminus, but also that helix TM1A is subject to enhanced mobility. TM1A moves towards the transporter core in the membrane environment, reaching a conformation that is closer to the structure of LeuT with wild type sequence, indicating that the mutation introduced to create the inward-facing structure might have altered the position of helix TM1A. The mobile N-terminus avoids entering the open vestibule of the inward-facing state, as accessibility studies do not show any reduction of quenching by iodide of a fluorophore attached to the N-terminus.

## Introduction

Neurotransmitter:sodium symporters (NSS) are secondary active transporters that belong to the solute carrier family 6 (SLC6), and include transporters for the neurotransmitters serotonin (SERT), dopamine (DAT), norepinephrine (NET) and γ-aminobutyric acid (GAT). The NSS transporters are pharmacologically relevant targets for the treatment of several neurological and psychiatric disorders [1], but are also the target of a number of illicit drugs.

The small amino acid transporter LeuT from *Aquifex aeolicus* is a bacterial homolog of NSS transporters that has been crystalized in three different conformations [2–4]. These have been interpreted as representing the outward-open [2], the outward-occluded [3] and the inward-open [4] conformation of the transport cycle. Most mammalian SLC 6 transporters consist of 12 transmembrane (TM) helices, where TM1-5 and TM6-10 form pseudo-symmetric inverted repeats [5]. Transporters of the SLC6 family use the electrochemical gradient of sodium, possibly also of chloride and potassium, to energize substrate transport [6, 7]. The SLC6 transporters operate according to the alternating access model [8], which states that the substrate binding site within the transporter can be accessed from both sides of the membrane, but at any given time the path to the substrate binding site is only accessible from one side of the membrane. This avoids the formation of a channel-like mode and also imposes directionality of transport, in the presence of pertinent ion gradients.

The dynamics of LeuT has been studied by electron paramagnetic resonance (EPR) [9, 10], solid state NMR [11], fluorescence resonance energy transfer microscopy (FRET) [12–16], lanthanide resonance energy transfer (LRET) [17] and also by molecular dynamics (MD) simulations [15–24]. The human monoamine transporters have been extensively studied with respect to efflux of substrate [25–28].

Regulation of efflux is organized via a number of different events which take place intracellularly: (i) intracellularly located kinases such as protein kinase C or CamKII alpha interact with the C-terminus to phosphorylate the N-terminus and thereby trigger efflux [29–32], (ii) constituents of the plasma membrane such as phosphoinositide phosphatidylinositol-4,5-bisphosphate (PIP2) bind to the monoamine transporters and sustain efflux [33–35]. Most recently, it has been shown that the amino termini of the dopamine transporter synergistically contribute to substrate and inhibitor affinities [36]. These regulatory events suggest a highly active contribution of especially the N-terminus to inward and outward transport processes.

In the current study, we investigated the dynamics of the N-terminus of LeuT to gain more insight into the degree of conservation between closely related but functionally distinct transporters with respect to defined regulatory functions. We combined molecular dynamics simulations with LRET-based distance measurements and iodide quenching of a fluorophore chemically linked to the N-terminus of LeuT. We found/identified a pronounced change in the dynamics of the N-terminus, contingent on the adopted conformational state. The N-terminus had a single conformation in the outward-facing state, while it was highly dynamic in the inward-facing state. It did not show any propensity to enter the open inner vestibule. The latter would prevent rebinding of substrate and thus hamper efflux.

## Materials and Methods

### Molecular Biology

The plasmid encoding LeuT was a kind gift from Eric Gouaux (Vollum Institute, OHSU—Oregon Health and Science University). The lanthanide binding tag (LBT) is a genetically encoded tag comprised of 17 amino acids with the sequence YWDTNNDGWYEGDELLA. The LBT tag was introduced in LeuT at extracellular loop 4 (EL4) between residue A335 and G336 or at the C-terminus between residues R519 and G520 using two successive PCR reactions. The first reaction was performed to generate mega primers (A335–LBT–G336 and R519–LBT–G520), which were purified from an agarose gel. The purified mega primers were used in a second PCR (quick change) to introduce the LBT encoding sequence between residue A335 and G336 as well as between residues R519 and G520. Cysteine mutants (K4C, H7C, A9C) in the background of the LeuT–A335–LBT–G336 were generated via site-directed mutagenesis using quick change PCR. All the mutants were subsequently confirmed by DNA sequencing. The primer sequence for all the mutants is listed below.

**Table:** 

LeuT LBT forward primer	GCCATATTCTCCCAAACGGCCTATTGGGATACCAACAACG
LeuT LBT reverse primer	AAATCCTAAAAAGGTTCCTCCCGCCAGCAGTTCATCGCC
LeuT K4C forward primer	CTTTAAGAAGGAGATATACCATGGAAGTTTGCAGGGAACACTGGGCG
LeuT K4C reverse primer	CGCCCAGTGTTCCCTGCAAACTTCCATGGTATATCTCCTTCTTAAAG
LeuT H7C forward primer	GATATACCATGGAAGTTAAAAGGGAATGCTGGGCGACGCGAC
LeuT H7C reverse primer	GTCGCGTCGCCCAGCATTCCCTTTTAACTTCCATGGTATATC
LeuT A9C forward primer	GAAGTTAAAAGGGAACACTGGTGCACGCGACTCGGTTTAATCCTC
LeuT A9C reverse primer	GAGGATTAAACCGAGTCGCGTGCACCAGTGTTCCCTTTTAACTTC
LeuT K4C/R5A forward primer	GTCGCGTCGCCCAGTGTTCCGCGCAAACTTCCATGGTATATCTCCTTCTTAAAG
LeuT K4C/R5A reverse primer	CTTTAAGAAGGAGATATACCATGGAAGTTTGCGCGGAACACTGGGCGACGCGAC
LeuT H7C/R5A forward primer	GAGTCGCGTCGCCCAGCATTCCGCTTTAACTTCCATGGTATATCTCCTTCTTAA
LeuT H7C/R5A reverse primer	TTAAGAAGGAGATATACCATGGAAGTTAAAGCGGAATGCTGGGCGACGCGACTC
LeuT A9C/R5A forward primer	AAACCGAGTCGCGTGCACCAGTGTTCCGCTTTAACTTCCATGGTATATCTCCTTCT
LeuT A9C/R5A reverse primer	AGAAGGAGATATACCATGGAAGTTAAAGCGGAACACTGGTGCACGCGACTCGGTTT
LeuT R30A forward primer	CGCAGCTTGAACGGGAAATGCGAGGAAATTACCAAGTCCT
LeuT R30A reverse primer	AGGACTTGGTAATTTCCTCGCATTTCCCGTTCAAGCTGCG
LeuT-LBT forward primer^a^	GCCATATTCTCCCAAACGGCCtattgggataccaacaacg
LeuT-LBT reverse primer^a^	AAATCCTAAAAAGGTTCCTCCcgccagcagttcatcgcc

^a^Upper case letters represent the 5′ overhang complementary to the LeuT cDNA, while the lower case letters represent the LBT complementary DNA sequence

### Expression and Purification of LeuT

Expression and purification of LeuT was carried out as reported by Yamashita et al. [3] with slight modifications. Briefly, wild type LeuT and LBT constructs of LeuT were expressed in commercial C41 (DE3) competent *E.* *coli* strains. Expression was induced with 0.2 M isopropyl-β-d-thiogalactopyranoside (Thermo Fisher) when OD600 reached 0.6. The induced culture was rotated in a shaker incubator for 20 h at 18 °C at 180 rpm. Cells were harvested and mixed with lysis buffer (glycerol 10%, NaCl 200 mM, HEPES 20 mM, pH 7.5, MgCl_2_ 5 mM, protease inhibitor cocktail 1 tablet per 100 ml, DNase-1 20 µg/ml, lysozyme 0.4 mg/ml, TCEP 0.5 mg/ml) and subjected twice to lysis with Emulsiflex C-3 (Avestin) under a pressure of 15,000 psi. The supernatant was separated from cell debris by centrifugation at 5000×*g* and subsequently placed into an ultracentrifuge for 2.5 h at 120,000×*g* to collect the crude membrane fraction. Subsequently, crude membranes were manually homogenized and solubilized in membrane solubilization buffer (NaCl 200 mM, HEPES 20 mM, pH 7.5, PMSF 1 mM, DDM 1%) for 1.5 h on a magnetic stirrer with low speed stirring at 4 °C. The non-solubilized fraction was removed by ultracentrifugation at 120,000×*g* at 4 °C for 30 min. The soluble membrane fraction was incubated overnight at 4 °C with pre-equilibrated TALON metal affinity resin (Takara). LeuT bound to the resin was eluted with elution buffer (NaCl 200 mM, HEPES 20 mM, imidazole 350 mM). Finally, the imidazole was removed using PD10 columns (GE Healthcare).

Labeling of cysteine with the fluorescent dye tetramethylrhodamine maleimide (TMR) was done using a molar LeuT to dye ratio of 1:3. The mixture was wrapped with aluminum foil and incubated at 4 °C for 3 h with gentle rotation on a multi rotator (PTR-30, Grant-bio) before LeuT was eluted from the metal affinity resin. The column was washed extensively to remove the unbound dye, and LeuT was subsequently eluted from the column. LRET-based measurements were always performed using freshly purified and labeled samples.

### Protein Reconstitution

Reconstitution of detergent-solubilized and TMR-labeled LeuT into POPC liposomes was carried out as described in [37] using a protein to lipid ratio of 1:100. Briefly, the organic solvent containing POPC lipids (Avanti) was dried under a gentle stream of argon. Traces of remaining solvent were removed overnight using a rotavapor. Dried lipid layers were dissolved in buffer (KCl or NaCl 200 mM, HEPES 20 mM, pH 7.5) to a final concentration of 20 mg/ml. Solubilized lipids were sonicated for 45 min in stepwise 15 min cycles and 5 min pauses at 4 °C. This suspension was snap frozen with liquid nitrogen and thawed on ice to generate large multi lamellar vesicles (LMVs). The LMVs were extruded 13 times through an extrusion chamber (Avanti) using a filter of 400 nm pore size to create a homogenized population of LMVs. The membranes of these liposomes were destabilized using Triton-X100 detergent. LeuT was reconstituted at a protein to lipid ratio of 1:100 and incubated for 30 min at 4 °C. Detergent was removed by incubating the liposomes with Bio-Beads SM-2 (BIO-RAD) followed by ultracentrifugation at 120,000×*g* for 80 min. Proteoliposomes were collected and re-suspended in the buffer (KCl or NaCl 200 mM, HEPES 20 mM, pH 7.5) to a final concentration of 100 mg/ml and freshly used or stored at − 80 °C until use.

### [3H] Leucine Binding

The scintillation proximity assay (SPA) was performed to examine whether LeuT and the LeuT-mutants were correctly folded. Binding activities of the wild type and mutant transporters were measured in a competitive binding assay of cold and hot leucine. [3H] leucine has an activity of 54.1 Ci/mmol (PerkinElmer). Copper coated yttrium silicate YSi (PerkinElmer) beads were used to bind the His-tagged LeuT as described in [38].

### [3H] Alanine Uptake

[3H] alanine uptake experiments were performed as described in [39]. LeuT containing proteoliposomes were created, which differed in the type of internal buffer (KCl 200 mM, HEPES 20 mM, pH 7.5) and external buffer (NaCl 200 mM, HEPES 20 mM, pH 7.5). Uptake of [3H] alanine (specific activity 84.1 Ci/mmol (PerkinElmer)) was initiated by loading the proteoliposomes into external buffer using the indicated concentration of [3H] alanine at 27 °C. The reaction was terminated by adding 10-fold excess of ice cold external buffer. Proteoliposomes were collected on a 0.22 µm pore sized nitrocellulose filter paper GSWP (Merck Millipore Ltd.). The filter papers were washed 3 times with ice cold external buffer and vacuum dried. Scintillation cocktail (2 ml) was added to the dried filter papers and mixed for at least 2 h before radioactivity was measured in a liquid scintillation analyzer (Tri-carb 2800TR, PerkinElmer). Analyses were performed using non-linear regression with GraphPad Prism 5.0.

### LRET Measurements

The genetically encoded lanthanide binding tag (LBT) was introduced between residues A335 and G336 at the extracellular side of LeuT and between residues R519 and G520 at the C-terminus of LeuT. This tag encages terbium (Tb^+3^), which acted as the donor fluorophore. The TMR dye was chemically linked using maleimide chemistry to cysteines at the N-terminus (residue K4C, H7C, A9C) and served as the acceptor fluorophore.

Donor emission in the presence and absence of the acceptor was recorded as described in [17]. Tb^+3^ emits light at 4 different wavelengths (λ 490 nm, 545 nm, 585 nm and 620 nm). The Tb^+3^ emissions were recorded using a long pass filter.

Data were analyzed by fitting the decays of the Tb^3+^ emission in the absence or presence of the acceptor fluorophore (TMR). Donor decays were fitted in MATLAB using the sum of three exponentials, as described in [17]. The fastest decaying component is an instrument artefact and constant for all measurements, the intermediate component is linked to protein aggregation, while the slowest component reports on the distance between the donor and the acceptor probe. The Förster equation (R = R_0_(π_DA_/(π_D_ _−_ π_DA_))^1/6^) was used to calculate intramolecular distances, where R is the donor to acceptor distance, R_0_ is Förster distance, τ_DA_ is the donor decay in the presence of the acceptor fluorophore and τ_D_ is the donor decay in the absence of the acceptor. Measurements were carried out in the presence of a high Na^+^ concentration (200 mM), the bound substrate alanine and the inhibitor tryptophan, respectively. Furthermore, inner and outer salt bridges (R5A, R30A) were disrupted to lock the transporter in an inward-open or outward-open conformation, respectively. All measurements were performed in detergent (DDM).

### Protein Labeling and KI Quenching of Fluorescence

LeuT was labeled with TMR utilizing a protein to dye ratio of 1:3. The reaction tube containing protein and dye was wrapped in aluminum foil and placed into a Multi-Rotator (PTR-30, Grant-bio), where the labeling reaction was allowed to proceed for 3 h at 4 °C. Samples were then mixed with talon metal affinity resin for HiS-tag binding and washed with 5 column volumes of buffer to remove the excess dye and eluted with 350 mM imidazole. Imidazole was removed by using PD-10 columns (SephadexTM G-25 M, GE Healthcare). Protein was quantified and 0.5 µg per ml of labeled protein samples were diluted in high sodium buffer (NaCl 200 mM, HEPES 20 mM, pH 7.5 and DDM 0.05%). Cysteine accessibility experiments as specified in Billesbølle et al. [40] were recorded on an HITACHI (F-4500) fluorescence spectrophotometer at λ_em_ = 572 nm by an excitation source at λ = 540 nm and excitation/emission band passes of 5 nm at room temperature. Emission spectra were recorded in the λ_em_ range between 550–650 nm and in 1 nm increments. Aliquots of the quencher KI were added sequentially and the fluorescence intensities recorded. Fluorescence intensities (F) were corrected for sample dilutions and normalized to the initial fluorescence intensities in the sample (F_0_) in the absence of the quencher KI. Data were analyzed by linear regression in GraphPad Prism v5.0.

The Stern–Volmer equation F_0_/F = 1 + KSV × [Q] was used to measure the extent of accessibility, where F_0_/F is normalized fluorescence quenching, KSV is the Stern–Volmer constant and [Q] is the concentration of the quencher KI. Spectra were recorded at a sampling rate of 10 s^−1^ under continuous stirring with a magnetic stirrer inside the cuvette, which contained 0.5 µg per ml protein sample labeled with TMR in a high sodium buffer (NaCl 200 mM, HEPES 20 mM, pH 7.5 and DDM 0.05%).

### In Silico Model Building, Simulation Setup and Parameters

Simulations used the inward-facing and the outward-occluded structure of LeuT (PDB ID: 3TT3 and 2A56) [3, 4] using residue 5 to 515 as were described in [17]. In short, missing residues of LeuT models were built using MODELLER version 9.12 [41], mutations reverted to wild type, sodium ions were added where missing. The missing residues of the N-terminus of the inward-facing conformation were copied from the outward-occluded LeuT structure, following super positioning of helix TM1A. The inward-facing structure was simulated in palmitoyl-oleoyl-phosphatidyl-choline (POPC) containing membranes and in n-octyl-β-d-glucopyranoside (BOG) micelles, while the outward-occluded model was tested in membrane only. For the membrane simulations, LeuT was inserted into a pre-equilibrated membrane consisting of 174 POPC lipid molecules using the membed method as described earlier in [42]. The micelle structure was created using a self-assembly procedure using 140 BOG molecules, while restraining LeuT 1000 kJ/mol/nm^2^ in 100 ns long simulations. Systems were electro-neutralized and an ionic concentration of 150 mM NaCl was added. Each system was then equilibrated for a total of 30 ns, while slowly releasing the transporter by reducing the position restraints on the heavy atoms of LeuT in three steps: 1000, 100 and 10 kJ/mol/nm^2^. Production runs were carried out for 200 ns with the Gromacs simulation package version 4.5.4 [43]. Berger lipids [44] were used to describe the POPC membrane. The OPLS force field [45] was used to describe the protein and detergent. Electrostatic interactions were treated using the particle mesh Ewald summation method with a cutoff of 1.0 nm. The Berendsen semi-isotropic pressure coupling scheme was applied for the membrane simulation and isotropic pressure coupling for the micelle simulations at 1 bar in both cases [46]. Temperature was maintained at 310 K using the v-rescale (τ = 0.1 ps) thermostat [47].

## Results

**Fig. 1:**
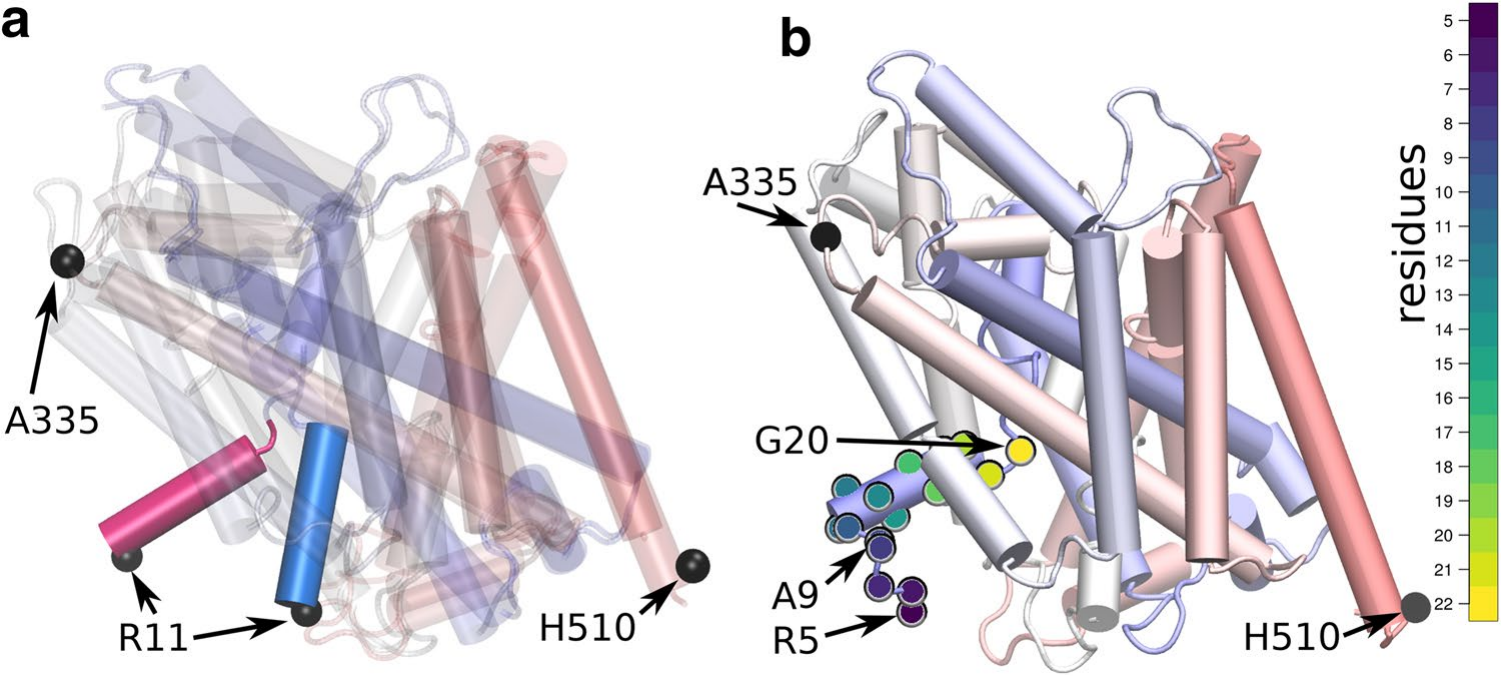
Movements of TM1A. **a** Inward-facing and outward-facing LeuT are overlayed on the scaffold domain. Helix TM1A is highlighted in red for the inward-facing conformation and blue for the outward-facing conformation. The Cα atoms of residue R11 on helix TM1A and sites of LBT insertion are shown as black spheres. **b** Structure showing the Cα atoms of residues on the N-terminus and helix TM1A. Distances distribution to residue A335 preceding helix TM8 and to residue H510, the last stable helical residue of helix TM12 are shown in Fig. 2

Crystal structures of LeuT have been solved in the outward-open, the outward-occluded and the inward-open conformation [2–4]. For the inward-facing conformation, these structures (Fig. 1) show that helix TM1A moved upwards into a region that would be occupied by lipids in the cell membrane. Previously, by simulations and LRET measurements, we have shown that this conformation of helix TM1A is unstable [17] in the membrane environment, but stable in the micelle environment. Importantly, conformational changes allow for the charged residues of the N-terminus and of helix TM1A to partition out of the membrane. Here, we continued this investigation to determine the extent by which helix TM1A and the preceding N-terminal residues move. We therefore compared the results of simulations for the outward-facing state with that of the inward-facing state in the membrane and in a detergent micelle, respectively.

### Mobility of the N-Terminus

**Fig. 2:**
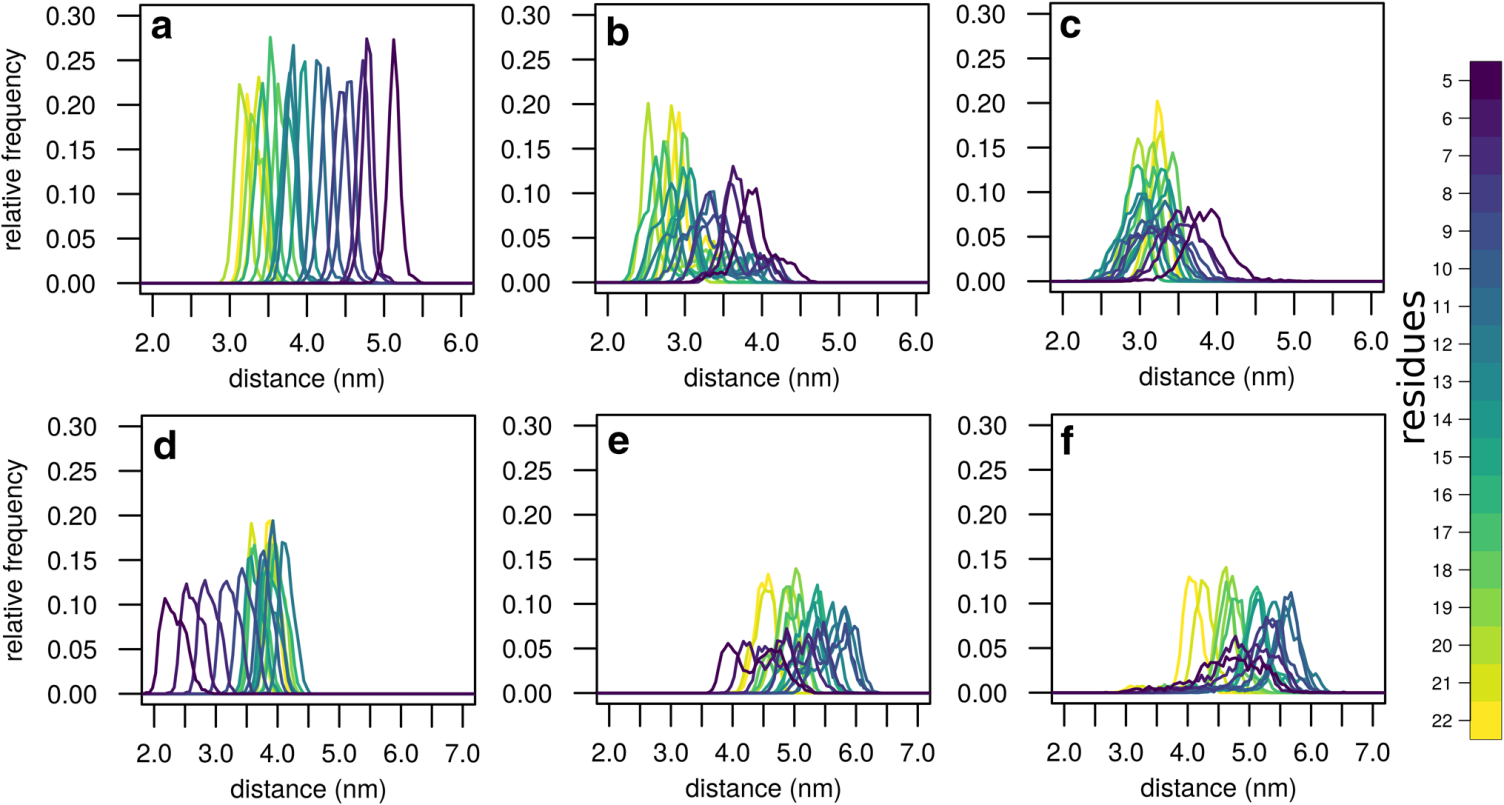
Distributions of distances to the first 22 residues of LeuT. The upper row shows distances between the Cα of the first 22 residues to the Cα atom of residue G335 for **a** the membrane inserted outward facing LeuT, **b** membrane inserted and inward-open LeuT and **c** micelle solubilized and inward-open LeuT. The lower row shows distances of the first 22 residues to the Cα atom of residue H510, which is the last helical residue of TM12, for **d** the membrane inserted outward-facing LeuT, **e** membrane inserted and inward-open LeuT and **f** micelle solubilized and inward-open LeuT

The crystal structures of LeuT show a much shorter distance between the helix TM1A and the extracellular loop 4. The distance between residue R11 on TM1A and residue A335 in the extracellular loop 4 is 2.96 nm in the inward-facing conformation but 4.19 nm in the outward-facing conformation, reflecting the large movement. Simulations confirmed the difference in distance, but also revealed larger differences. To quantify these structural changes, we measured the distance between the Cα atom of residue A335 and the Cα atoms of the first 20 residues of LeuT (the N-terminus and helix TM1A). Overall, the distances were longer for the outward-facing state (Fig. 2a) than for the inward-facing state (Fig. 2b, c). The outward-facing state showed sharp distance distributions. A single conformation is consistent with a well-defined geometry at the intracellular side. In contrast, distributions of distances characterize the inward-facing state in the membrane (Fig. 2b) and the micelle environment (Fig. 2c). For the inward-facing conformation, the distributions of distances of residue A335 to the first 20 residues of LeuT (Fig. 2b, c) differed between the membrane and the micelle environment. The distances for helix TM1A were shorter for the membrane environment compared to micelle environment. In the membrane environment we observed that helix TM1A moves towards the core of LeuT, shortening the distance between helix TM1A and TM1B to values observed for the outward-facing conformation. In contrast, helix TM1A did not move in the micelle environment, consistent with the conformation of the crystal structure (PDB ID: 3TT3) [4]. From these observations we infer that the conformation of TM1A observed by crystallography is compatible with the micelle environment, but not with a cell membrane. Shortening of the TM1A–TM1B distance is most likely a consequence of using the wild type sequence of LeuT, while the inward-facing conformation of LeuT required two mutations in substrate binding site 1 (S1) leading to TM1A dislocation. The larger movement of helix TM1A in the membrane as compared to the micelle environment is a direct consequence of the membrane environment. Helix TM1A repartitions out of the membrane, driven by the charged residues at the N-terminal end of helix TM1A, thereby also leading to the reduction of the TM1A–TM1B distance.

The residues at the N-terminus preceding helix TM1A show comparable distances to residue A335 and large distributions, indicating that the N-terminus is unconstrained in the inward-open conformation. Therefore, while being in a single conformation in the outward-facing state, the N-terminus is highly dynamic in both the membrane and the micelle environment, which leads to the broad distributions of inter-residue distances.

A similar pattern appeared when measuring movements parallel to the membrane by detecting distances from helix TM12 to the N-terminus and helix TM1A (Fig. 2d–f). We found sharp distributions for the outward-facing conformation that become a bit broader towards the N-terminus, reflecting higher mobility (Fig. 2d). In the inward-facing conformation, the distributions observed for helix TM1A were shifted towards larger distances and were broader. The distances were larger in the micelle environment (Fig. 2f) compared to the membrane environment (Fig. 2e), especially for the beginning of helix TM1A. This change is a consequence of the movement of helix TM1A away from the C-terminus into the membrane and towards the extracellular side. The first residues preceding helix TM1A show much broader distributions and therefore larger mobility compared to helix TM1A. As observed for distances to residue A335, the distribution is wider in the micelle, consistent with enhanced movements due to the environment. Interestingly, the mobility is much larger parallel to the membrane (as measured to residue H510 in helix TM12 of LeuT) than vertically to the membrane (as measured to A335 in EL4). This result implies that the N-terminus shows larger movements and dynamics parallel to the membrane plane than vertical movements, also if LeuT is in a micelle environment.

**Fig. 3:**
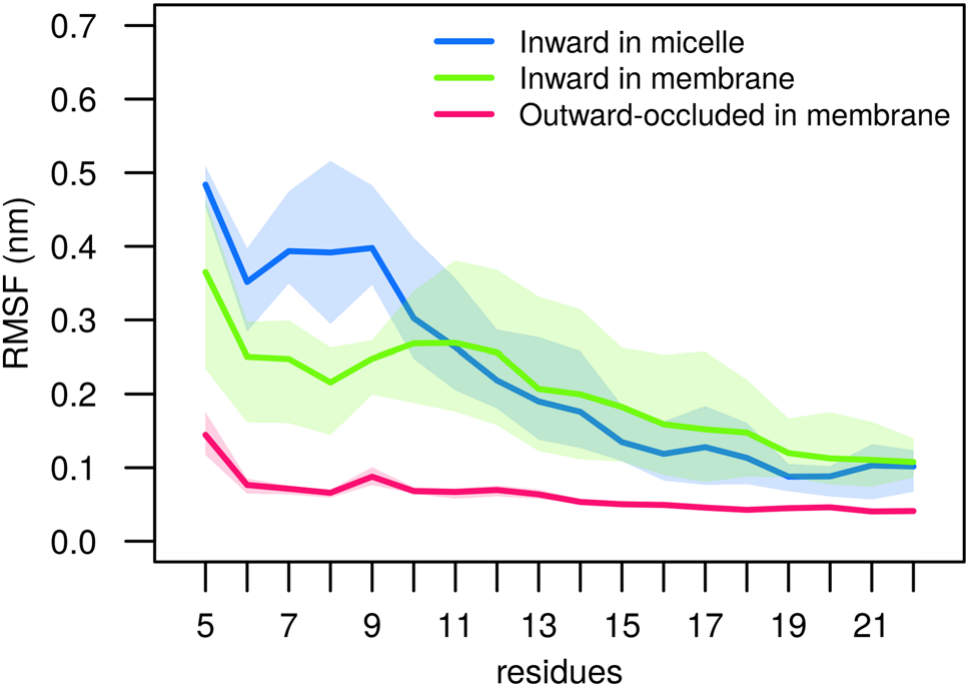
Flexibility of the N-terminus. The RMSF of residue 5 to 22 shows the mobility of the N-terminus and helix TM1A

To further characterize the dynamics of the N-terminus and helix TM1A, we measured their root mean square fluctuations (RMSF) (Fig. 3). Consistent with the broader distribution of distances (Fig. 2) for the inward-facing state, we found that the mobility of the inward-facing conformation was higher than that of the outward-facing conformation in which the N-terminus adopts a single conformation. For the inward-facing conformation, fluctuations showed two amplitude regimes: (i) Helix TM1A showed the smaller mobility regimen, because it has a helical secondary structure and is more restrained by interactions with the transmembrane core of LeuT (ii) as compared to the preceding N-terminus, which is no longer restrained by the interactions of R5 with D369. The membrane environment restricts the mobility of the N-terminus as residue 5–9 showed smaller RMSF values. The membrane contributes indirectly to the reduced RMSF by limiting the available space as compared to the micelle environment. The membrane forms an extended flat barrier, which the highly polar N-terminus cannot enter.

### Inserting an LBT Tag into the Extracellular Loop 4

To experimentally measure distances between the extracellular side and residues before helix TM1A, we inserted a lanthanide binding tag (LBT) into the loop connecting extracellular loop 4 (EL4) and helix TM8, between residues A335 and G336.

**Fig. 4:**
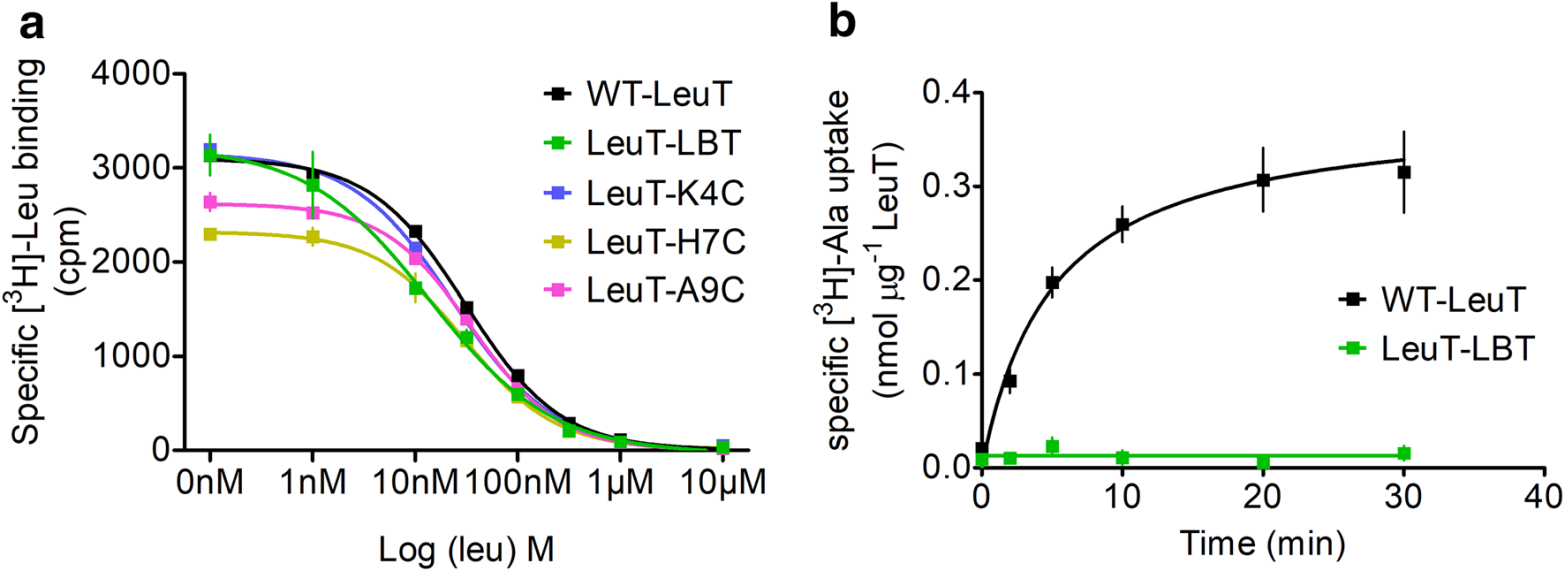
Inserting the LBT into the EL4. **a** Competitive binding of cold leucine to [3H] leucine bound LeuT using the scintillation proximity assay of wild type LeuT and LeuT mutants with the LBT tag inserted in the EL4 loop between A335 and G336. **b** [3H] alanine uptake in neutral lipid (POPC) proteoliposomes of wild type LeuT and LeuT with the LBT tag inserted in the EL4. Uptake was carried out for 30 min at 27 °C, non-specific binding was subtracted from total uptake. All data are obtained from three independent experiments with error bars denoting standard deviation

We used the scintillation proximity assay to measure ligand binding to LeuT wild type and mutants to assure proper transporter folding (Fig. 4a). Wild type LeuT and the LBT carrying mutants showed indistinguishable affinities for leucine. The K_I_ for wild type LeuT was 15.5 ± 2.4 nM, for LeuT carrying the extracellular LBT = 12.0 ± 2.6 nM, while the single cysteine mutants of the LBT carrying LeuT were 11.8 ± 2.4 nM for LeuT-K4C, 15.4 ± 3.1 nM for LeuT-H7C, and 15.1 ± 1.7 nM for LeuT-A9C. These values agree with reported affinities of K_I_ of 16 ± 1 nM [2], confirming that LeuT is correctly folded and leucine binding competent.

**Fig. 5:**
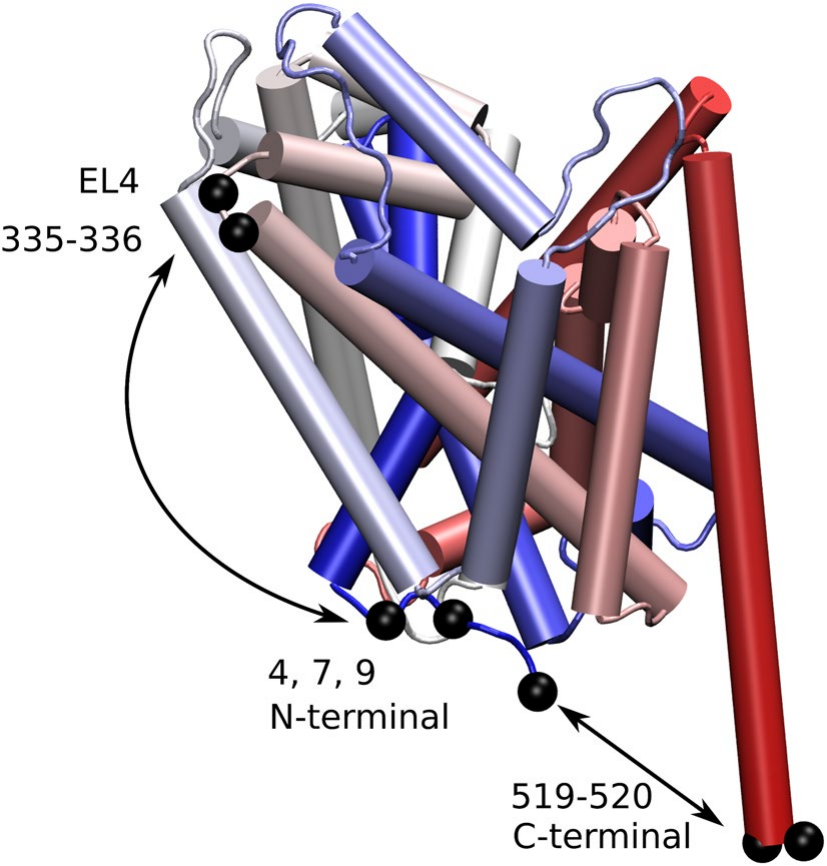
LBT tag positions. LeuT structure showing the position of the inserted LBT tags (EL4 and C-terminus) and the position of residues K4, H7 and A9

In contrast to substrate binding, inserting the LBT into EL4 abolished the transport capability of LeuT (Fig. 4b). While wild type LeuT reconstituted into liposomes showed normal uptake of alanine, the mutant transporter carrying the LBT did not. Taken together, these data suggest that inserting the LBT into the extracellular loop 4 creates a transporter which is properly folded, but locked in the outward-facing state. In contrast, when inserting the LBT tag into the C-terminus of LeuT (Fig. 5), we observed unchanged normal affinity for leucine and unaffected substrate uptake [17].

### Distance Measurement Using LRET

We used LRET based distance measurements, in which we employed two different LBT constructs to monitor movements of the N-terminus of LeuT: (i) one in which the LBT tag was at the C-terminus (this construct was transport competent) and (ii) one in which the LBT tag was inserted into EL4 (i.e. between residues 335 and 336). The latter construct bound substrate but failed to support substrate transport. The two constructs were designed to allow for distance measurements across and parallel to the membrane (Table 1).

**Table 1:** Distances between the Tb^3+^ bound to the LBT and TMR chemically linked to cysteine residues

Mutants	KCl (nm)	NaCl (nm)	NaCl Leucine (nm)	NaCl Alanine (nm)
K4C-extacellular LBT	8.63 ± 0.08	8.66 ± 0.17	8.70 ± 0.04	8.73 ± 0.04
K4C-extacellular LBT, R30A	7.98 ± 0.04	8.16 ± 0.07	8.21 ± 0.04	8.14 ± 0.08
K4C-extacellular LBT R5A	7.79 ± 0.15	7.96 ± 0.15	7.96 ± 0.15	7.97 ± 0.14
H7C-extacellular LBT	7.64 ± 0.02	7.74 ± 0.01	7.82 ± 0.08	7.81 ± 0.02
H7C-extacellular LBT, R30A	7.19 ± 0.04	7.26 ± 0.05	7.37 ± 0.03	7.33 ± 0.03
H7C-extacellular LBT, R5A	7.12 ± 0.16	7.22 ± 0.14	7.22 ± 0.14	7.20 ± 0.13
A9C-extacellular LBT	7.02 ± 0.01	7.08 ± 0.11	7.17 ± 0.00	7.18 ± 0.02
A9C-extacellular LBT, R30A	6.67 ± 0.03	6.83 ± 0.06	6.81 ± 0.03	6.82 ± 0.07
A9C-extacellular LBT, R5A	6.81 ± 0.07	6.95 ± 0.07	6.99 ± 0.05	6.83 ± 0.04
K4C-extacellular LBT		7.82 ± 0.24	7.37 ± 0.12	6.69 ± 0.35
H7C-extacellular LBT		7.98 ± 0.02	6.86 ± 0.51	6.82 ± 0.14
A9C-extacellular LBT		7.33 ± 0.02	7.29 ± 0.36	7.56 ± 0.21
K4C-intratacellular LBT		4.74 ± 0.28	4.46 ± 0.16	4.53 ± 0.01
H7C-intracellular LBT		5.11 ± 0.36	4.32 ± 0.07	4.33 ± 0.17
A9C-intracellular LBT		5.16 ± 0.22		

We found a consistent picture of distances for the fluorophore TMR attached to residues K4C, H7C and A9C using the LBT tag at the extracellular site. The distance for residue A9C was shortest, while the distance for residue K4C was the longest. The measured distances were unaffected by replacing sodium with potassium or by adding the substrate alanine or the inhibitor tryptophan (Trp), respectively. The inhibitor Trp is known to bind to the outward-facing conformation of LeuT [2]. To confirm that the LBT tag inserted into the EL4 trapped LeuT in the outward-facing conformation, we mutated the salt bridges of the inner gate (i.e. R5) and of the outer gate (i.e. R30). The R30A mutant stabilizes LeuT in the outward-facing state by preventing closure of the outer gate. The R5 mutant is inward-facing, because with the inner gate destroyed the outward-facing state was destabilized. Importantly, all distances measured with the external LBT were consistent with the outward-facing conformation, regardless of whether they were determined in the R5A or the R30A background. This confirms that the LBT tag inserted into EL4 locks LeuT in the outward-facing conformation.

In contrast to this locked state, insertion of the LBT-tag into the C-terminus does not affect dynamics or function of LeuT. We found that the distance between residue A9C and the C-terminus was strongly dependent on the conformational state of LeuT. The distance was 6.26 ± 0.21 nm in the inward-facing state and 5.16 ± 0.22 nm in the outward-facing state [17]. These distances were longer when measured in detergent micelles as compared to those determined in membranes. This difference in distances provides information on how the membrane environment impinges on conformation and structural changes of LeuT.

In the presence of NaCl, which stabilizes LeuT in the outward-facing state [15], we found a distance of 5.16 ± 0.22 nm to residue A9C, 5.11 ± 0.36 to residue H7C and the shortest distance 4.74 ± 0.28 to residue K4C.

### Water Exposure of the N-Terminus

**Fig. 6:**
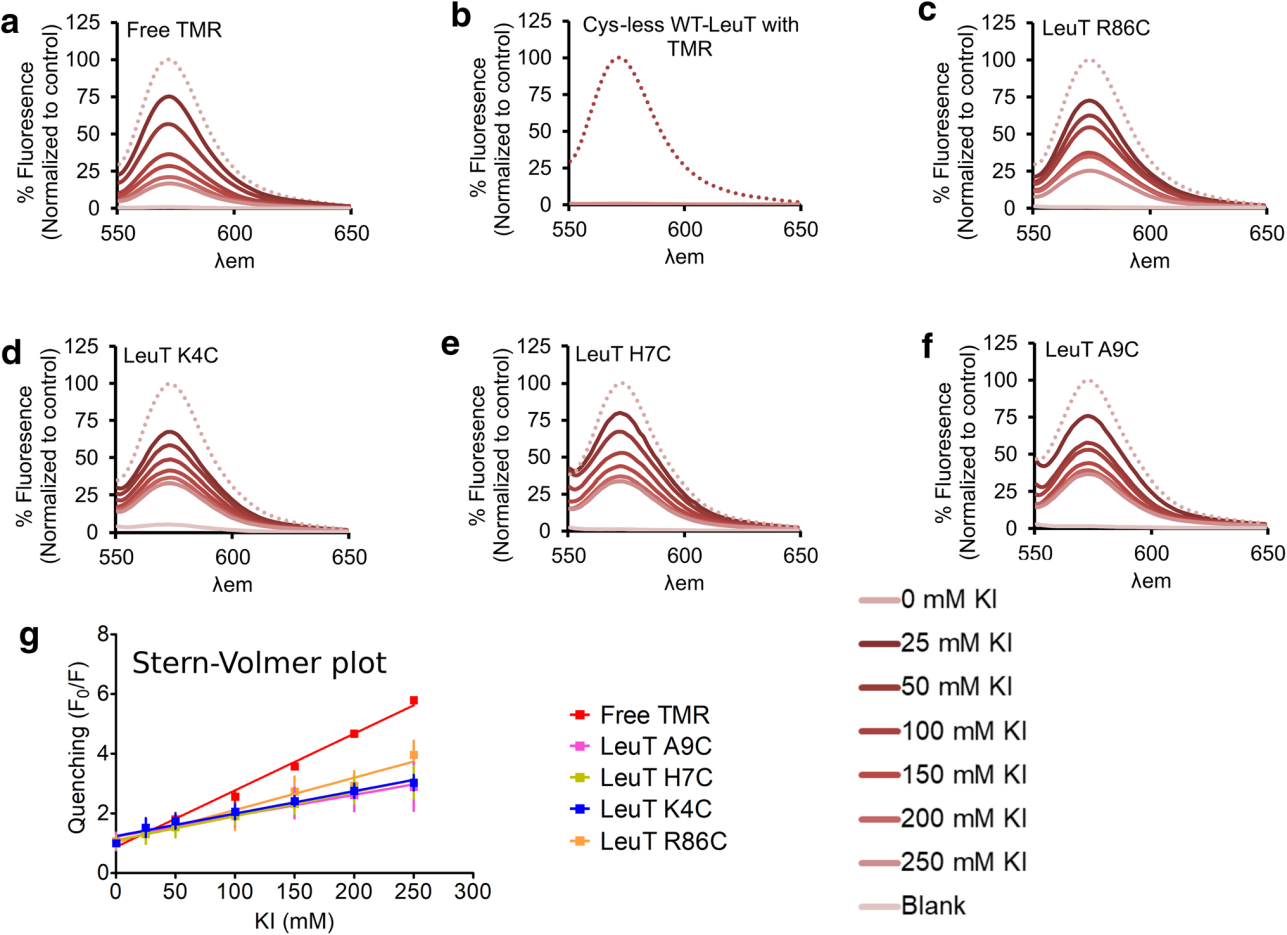
Cysteine accessibility and Stern–Volmer plot Iodide quenching. **a** shows fluorescent signal of free TMR and purified cysteine free LeuT. Panels **b-f** show the decrease of fluorescence signal with increasing concentration of the fluorescence quencher KI. **b** shows free TMR, panels **c-f** show traces for single cysteine variants of LeuT carrying the LBT tag at EL4 for the cysteine mutant for **c** R86C, which is conformation insensitive and water exposed, **d** R4C, **e** H7C, and **f** A7C. **g** Shows the Stern–Volmer plot of fluorescence quenching

Simulations for the inward-facing conformation showed that the mobility gained by the N-terminus leads to a structural ensembles, where its residues remain on average close to the transmembrane transporter core and could therefore potentially enter the open inner vestibule. To quantify the extent of water exposure of residues within the N-terminus, we relied on iodide quenching of fluorophores attached to cysteines within the protein. Collision of iodide with the fluorophore relaxes excited fluorophore to the ground state without emitting a photon. As reaching into the inner vestibule is associated with reduced exposure to iodide, we chemically linked TMR using maleimide chemistry to the single cysteine mutant of LeuT (K4C, H7C and A9C). We found that the fluorescence signal of TMR conjugated to the LeuT mutants was equally quenched at the tested positions and comparable to quenching measured with the control residue R86C, which is known to be water exposed and insensitive to conformational changes (Fig. 6). The Stern–Volmer plot (F_0_/F) (Fig. 6g), where F_0_ is the fluorescence without quencher and F is the fluorescence with quencher, shows that an increase in iodide concentrations yields a linear and overlapping dose dependence for all three N-terminal mutants. We note that unconjugated or free TMR also shows a linear dependency, but the slope is slightly steeper. Together these data show that residues 4, 7 and 9 are equally water accessible, rejecting the hypothesis that the N-terminus can enter the inner vestibule.

## Discussion

The prokaryotic small amino acid transporter LeuT uses the alternating access mechanism to transport substrate across the lipid bilayer [2–5, 9, 40, 48–50]. LeuT is sodium bound and outward-facing [15] in the resting state. Substrates bind with high affinity to the central substrate binding site S1, which is reachable through the open outer vestibule. Substrate binding triggers conformation changes, which lead to the closure of the outer vestibule and the opening of the inner vestibule, thereby forming the path for substrate release into the cytosol.

Our current study was motivated by the differences in function of the cytosolic termini between bacteria and mammals. In contrast to the membrane core of the transporters, the termini are not conserved throughout evolution in sequence, size and function, also between close paralogs such as the human serotonin and the dopamine transporters, which have very different sequences for their N-terminus. Mammalian monoamine transporters are subject to extensive phosphorylation, which leads to changes in their activity pattern [51], especially when looking at psychostimulant action [25]. Such phosphorylation sites are absent in the sequence of LeuT. Also, the intracellular termini of the mammalian transporters are important determinants for cell surface expression: (i) the N-terminus plays a role in the expression at the surface of the plasma membrane [52, 53], (ii) however, the C-terminus serves as the contact point for SEC24 proteins of the COPII coat complex [54], which supports trafficking to the surface of the plasma membrane [55–59]. A number of additional proteins may also interact with the intracellularly located termini. Of note, flotillin-1 [60, 61] and the plasma membrane-associated GTPase Rit-2 [62, 63] directly interact with monoamine transporters: They may also serve as functional regulators by changing the conformational ensemble of the termini, which behave like intrinsically disordered protein [64] or at the very least as highly dynamic protein parts [65]. The molecular details of the functional regulation is not well understood, because of the lack of sufficient high resolution structural knowledge, while it is well established that the intracellular termini of mammals have direct impact on the handling of the physiological substrates [36].

In contrast to the high variability of sequence and length of the N-terminus, a highly conserved arginine residues preceding helix TM1A by 5 residue is essential for SLC6 transporter function. It forms a salt bridge to an aspartate or glutamate at the end of TM8, thereby stabilizing the outward facing conformation by keeping the inner vestibule closed. In the serotonin transporter, residue R79 forms this salt bridge, in the dopamine transporter the respective residue is R60. In LeuT, it is residue R5 that stabilized the closed inner vestibule by a firm salt bridge with residue D369. This salt bridge is mirrored by the salt bridge at the extracellular gate [3, 66, 67], in LeuT it is formed between residue R30 and residue D404. Mutating one of the two aspartates of the inner or outer gate, also by a very conservative mutation such as glutamate, is sufficient to hamper transport. However, it was possible to regain transport function by mutating both aspartates to glutamates, which confirmed that these interactions are critical for maintaining the conformational equilibria in SLC6 transporters [66], and that they are interconnected by a seesaw-like mechanism.

Here, we investigated conformations and dynamics of the N-terminus of LeuT. We find that it is fully restrained in the outward-facing state, but that it is rendered highly flexible once the salt bridge between residue R5 and D369 opens in the inward-facing state. Our results from simulations, LRET measurements and iodide quenching suggest that the N-terminus is freely moving in the inward-facing conformation, while interactions with IL4 were suggested for GAT1 [68]. Distance distributions obtained from simulations suggested the possibility that the N-terminus could also access the open inner vestibule after substrate release and thereby block substrate efflux. To investigate this possibility, we use the fact that the N-terminal residues are expected to become less accessible upon entering the inner vestibule. Structural predictions indicated that residues 4 and 7 could potentially reach the vestibule, while residue 9 precedes the N-cap residue of helix TM1A and therefore cannot span the required distance. Accessibility studies using iodide quenching showed no difference between residues 4, 7 and 9. We therefore conclude that the N-terminus does not enter the inner vestibule. Previous studies on SERT showed that the truncation of the N-terminus affected amphetamine induced substrate release [69]. Our results on LeuT suggest that the N-terminus does not interfere with substrate release by blocking the intracellular vestibule and does not mirror this function of SERT. Instead we speculate that the N-terminus might shape the affinity of sodium to the inward-facing state, because the sodium concentration in the cytosol is a critical determinant of substrate release [70]. Small changes in intracellular sodium affinity may conceivably change the propensity of reverse transport, because dependent on the intracellular sodium activity for triggering efflux.
